# Supplementary material for: Use of Slaughterhouses as Sentinel Points for Genomic Surveillance of Foot-and-Mouth Disease Virus in Southern Vietnam
Source: Viruses. 2021 Nov 2;13(11):2203. doi: 10.3390/v13112203 (PMC8624567; doi:10.3390/v13112203)
Supplement: Supplementary file 1 [file viruses-13-02203-s001.zip › viruses-1371979-supplementary.pdf]

Table S1: Serology and OPF screening from farms in each province in 2015

Province	Serum Tested	Serum positive	Seropositive percentage of samples	OPF Screened (rRT-PCR)	OPF positive for FMDV RNA	Percentage OPF positive
Hà Tĩnh	500	86	17.2 (13,21)	112	0	0
Lạng Sơn	484	20	4 (2,5)	33	0	0
Phú Thọ	500	50	10 (7,12)	62	0	0
Bắc Kạn	*na*	*na*	*na*	*na*	*na*	*na*
Ninh Thuận	*na*	*na*	*na*	*na*	*na*	*na*
Đồng Tháp	485	149	31 (26,34)	139	36	25.8
Bình Phước	514	84	16 (13,19)	80	2	2.5
Đăk Lăk	504	142	28 (24,32)	142	3	2.1
TOTAL	2,987	531		568	41	7.2

Table S2: Serology and OPF screening from farms in each province in 2016

Province	Serum Tested	Serum positive	Seropositive percentage of samples	OPF Screened (rRT-PCR)	OPF positive	Percentage OPF positive
Hà Tĩnh	*na*	*na*	*na*	*na*	*na*	*na*
Lạng Sơn	236	31	13 (8,17)	33	2	6
Phú Thọ	253	34	13 (9,17)	32	2	6.2
Bắc Kạn	496	167	34 (29,37)	165	32	19.4
Ninh Thuận	250	177	71 (65,76)	177	20	11.2
Đồng Tháp	254	100	39 (33,45)	102	33	32.3
Bình Phước	*na*	*na*	*na*	*na*	*na*	*na*
Đăk Lăk	*na*	*na*	*na*	*na*	*na*	*na*
TOTAL	1,489	509		509	89	17.4

Table S3: Serology and OPF screening from farms in each province in 2017

Province	Serum Tested	Serum positive	Seropositive percentage of samples	OPF Screened (rRT-PCR)	OPF positive for FMDV RNA	Percentage OPF positive
Hà Tĩnh	*na*	*na*	*na*	*na*	*na*	*na*
Lạng Sơn	316	92	29 (24,34)	92	0	0
Phú Thọ	177	82	46 (39,53)	77	0	0
Bắc Kạn	982	447	46 (42,48)	443	33	7.4
Ninh Thuận	585	467	80 (76,83)	461	49	10.6
Đồng Tháp	369	210	57 (51,61)	212	85	40
Bình Phước	*na*	*na*	*na*	*na*	*na*	*na*
Đăk Lăk	252	182	72 (66,77)	181	56	31
TOTAL	2,681	1,480		1,466	223	

Table S4: Serology and OPF screening from farms in each province in 2018

Province	Serum Tested	Serum positive	Seropositive percentage of samples	OPF Screened (rRT-PCR)	OPF positive for FMDV RNA	Percentage OPF positive
Hà Tĩnh	*na*	*na*	*na*	*na*	*na*	*na*
Lạng Sơn	351	65	19 (14,22)	65	1	1.53
Phú Thọ	149	48	32 (24,39)	48	0	0
Bắc Kạn	752	363	48 (44,51)	347	3	0.8
Ninh Thuận	319	257	81 (76,84)	256	3	1.1
Đồng Tháp	540	284	53 (48,56)	284	31	11
Bình Phước	*na*	*na*	*na*	*na*	*na*	*na*
Đăk Lăk	942	605	64 (61,67)	605	27	4.4
TOTAL	3,053	1,622		1,605	65	

Table S5: Serology and OPF screening from farms in each province in 2019

Province	Serum Tested	Serum positive	Seropositive percentage of samples	OPF Screened (rRT-PCR)	OPF positive for FMDV RNA	Percentage OPF positive
Hà Tĩnh	*na*	*na*	*na*	*na*	*na*	*na*
Lạng Sơn	*na*	*na*	*na*	*na*	*na*	*na*
Phú Thọ	117	55	47 (38,56)	55	0	0
Bắc Kạn	560	287	51 (47,55)	286	5	1.74
Ninh Thuận	136	109	80 (73,86)	109	0	0
Đồng Tháp	317	145	46 (40,51)	145	12	8.27
Bình Phước	*na*	*na*	*na*	*na*	*na*	*na*
Đăk Lăk	475	304	64 (59,68)	302	11	3.64
TOTAL	1,605	900		897	28	

Table S6: Slaughterhouse Serology and OPF sample collection summary Long An

Sampling Round	Serum Tested	Number of positive 3ABC	Percentage 3ABC ELISA positive	OPF Screened (rRT-PCR)	OPF positive for FMDV RNA	Percentage OPF positive
1	30	21	70.0 (56,86)	30	7	23.3
2	30	10	33.3 (16.5, 50)	30	2	6.6
3	28	4	14.3 (1.3,27)	28	0	0
4	30	11	36.7 (19,53)	30	1	3.3
5	32	15	46.9 (29,64)	32	4	12.5
6	30	10	33.3 (16.5,50)	30	4	13.3
7	30	7	23.3 (8.2, 38)	30	4	13.3
8	30	11	36.7 (19.5,53)	30	3	10
9	30	10	33.3 (16.5, 50)	30	4	13.3
10	24	13	54.2 (34.3, 74)	24	5	20.8
11	36	17	47.2 (31,63)	36	6	16.6
12	23	9	39.1 (19.2,56)	23	0	0
13	37	11	29.7 (15,44)	37	3	8.1
14	30	9	30.0 (13.6,46)	30	1	3.3
15	20	11	55.0 (33,76)	20	4	20
16	40	10	25.0 (11,38)	40	3	7.5
Grand Total	480	179	37.3	480	51	

Table S7: Slaughterhouse Serology and OPF sample collection summary Tay Ninh

Sampling Round	Serum Tested	Number of positive 3ABC	Percentage 3ABC ELISA positive	OPF Screened (rRT-PCR)	OPF positive for FMDV RNA	Percentage OPF positive
1	30	28	93.3 (84, 100)	30	9	30
2	30	14	46.7 (29,64)	30	6	20
3	30	21	70.0 (53,86)	30	7	23.3
4	30	10	33.3 (16.5,50)	30	5	16.6
5	30	14	46.7 (29,64)	30	7	23.3
6	30	7	23.3 (8,38)	30	1	3.3
7	30	18	60.0 (42,77)	30	5	16.6
8	30	21	70.0 (53,86)	30	4	13.3
9	30	8	26.7 (11,42)	30	5	16.6
10	30	9	30.0 (13,46)	30	1	3.3
11	30	17	56.7 (39,74)	30	7	23.3
12	30	15	50.0 (32,74)	30	2	6.6
13	30	11	36.7 (19.5, 53.8)	30	2	6.6
14	30	20	66.7 (49.8,83)	30	4	13.3
15	30	13	43.3 (25.6, 70)	30	2	6.6
16	30	23	76.7 (61,91)	30	4	13.3
Grand Total	480	277	57.7	480	71	

Table S8: Large cluster information of serotype O

Name	Type	Area	Dates detected	Species	MRCA	Number of sequences	Closest lineage	Within cluster GD
Cluster 1	Farm and Outbreak	North	2010-12-22 to 2014-07-14	Pig, Cattle, Buffalo	2008.6 (1998.7,2020)	54	O/ME-SA/Pan Asia	0.028
	Farm and Outbreak	Central	2010-12-26 to 2012-05-23	Pig, Buffalo				
	Farm and Outbreak	South	2010-12-21 to 2017-01-09*	Pig, Cattle, Buffalo				
Cluster 2†	Farm and Outbreak	North	2017-10-01 to 2018-11-11	Pig, Cattle, Buffalo, Goat	2015.9 (2013.4,2019.6)	90	O/ME-SA/Pan Asia	0.011
	Outbreak	Central	2018-04-13	Cattle				
	Farm and Slaughterhouse	South	2017-01-10 to 2018-07-01	Cattle, Buffalo				
Cluster 6†	Outbreak	North	2018-11-13 to 2019-01-15	Pig	2017.9 (2017.5, 2019.1)	21	Mya-98	0.021
	Outbreak	Central	2018-12-27 to 2019-01-05					
	Outbreak	South	2018-11-13 to					
Cluster 8	Outbreak	North	2015-10-30 to 2017-09-23*	Pig, Cattle	2011.7 (2006.6,2020.9)	12	O/ME-SA/Ind2001d	0.021
		South	2015-09-04 to 2015-10-22	Pig, Cattle, Buffalo				
Cluster 9†	Outbreak	North	2016-01-16 to 2016-11-18	Cattle	2013.1 (2007.2,2019.2)	13	Mya-98 B	0.066
	Outbreak	Central	2013-10-07 to 2016-10-14	Pig (2016-11-18)				
	Farm and Slaughterhouse	South	2016-08-18 to 2017-12-15					

Cluster 10†	Outbreak	North		2016-11-16	Cattle Pig (2015-09-10)	20 09.7 (2002.9,2018.8)	22	O/ME-SA/Pan Asia	0.027
	Farm, Outbreak and Slaughterhouse	South	2013-05-17 to	2017-08-01					

† Clusters that were circulating in southern Vietnam during period of slaughterhouse sampling

* 2017 sequence does not cluster with other sequences in cluster 1 and 8, thus this clusters were not considered to be circulating after 2012 and 2015.

Table S9: Large cluster information for serotype A

Name	Type	Area	Dates detected	Species	MRCA	Number of sequences	Closest lineage	Within cluster GD
cluster9	Farm	North	2017-08-25 to 2018-01-08	Cattle	2015.3 (2012.2, 2019.5)	56	Sea/97	0.01
	Farm and outbreak	Central	2017-01-08 to 2019-01-05					
cluster5†	Outbreak	North	2017-08-29 to 2017-09-11	Cattle	2015.8 (2013.1,2019.5)	20	Sea/97	0.01
	Farm and outbreak	Central	2017-01-08 to 2017-08-05					
	Slaughterhouse	South	2017-10-17 to 2018-03-29					
cluster4†	Outbreak	North	2013-12-01 to 2014-04-24	Cattle Pig (2014-04-24)	2012.4 (2006.4,2019.4)	21	Sea/97	0.02
	Outbreak	Central	2013-10-09 to 2016-10-13	Cattle Pig (2015-09-10)				
	Outbreak and Slaughterhouse	South	2017-10-31 to 2019-06-01	Buffalo				
cluster10†	Farm	South	2017-01-06 to 2019-06-01	Cattle	2016.8 (2015.5,2020.1)	12	Sea/97	0.04
	Slaughterhouse		2018-01-24 to 2019-02-27					

† Clusters that were circulating in southern Vietnam during period of slaughterhouse sampling

Table S10: Model selection results from the Bayesian Evolutionary Sampling Tree (BEAST) where the best model, relaxed clock coalescent sky grid model was selected.

	Path sampling	Path Sampling average	Steppingstone sampling	Steppingstone sampling average
**Serotype O**				
Relaxed clock exponential	-8507.962087	-8507.037632	-8510.304413	-8509.431206
	-8506.113178		-8508.558	
Relaxed clock expansion	-8508.172778	-8508.342028	-8508.451305	-8508.291626
	-8508.511278		-8508.131947	
Relaxed clock constant	-8458.92312	-8458.449227	-8459.655856	-8459.274212
	-8457.975333		-8458.892568	
Relaxed clock coalescent sky grid	-8137.909934	-8138.998022	-8138.659178	-8139.871078
	-8140.086109		-8141.082978	
**Serotype A**				
	Path sampling	Path Sampling average	Steppingstone sampling	Steppingstone sampling average
Relaxed clock exponential	-5696.304571	-5695.588948	-5696.86083	-5696.043596
	-5694.873326		-5695.226363	
Relaxed clock expansion	-5697.271664	-5698.113582	-5698.07718	-5698.947185
	-5698.955501		-5699.817189	
Relaxed clock constant	-5706.882944	-5693.61778	-5706.951335	-5706.906472
	-5680.352616		-5706.86161	
Relaxed clock coalescent sky grid	-5686.396487	-5683.374551	-5688.637852	-5685.418112
	-5680.352616		-5682.198372	

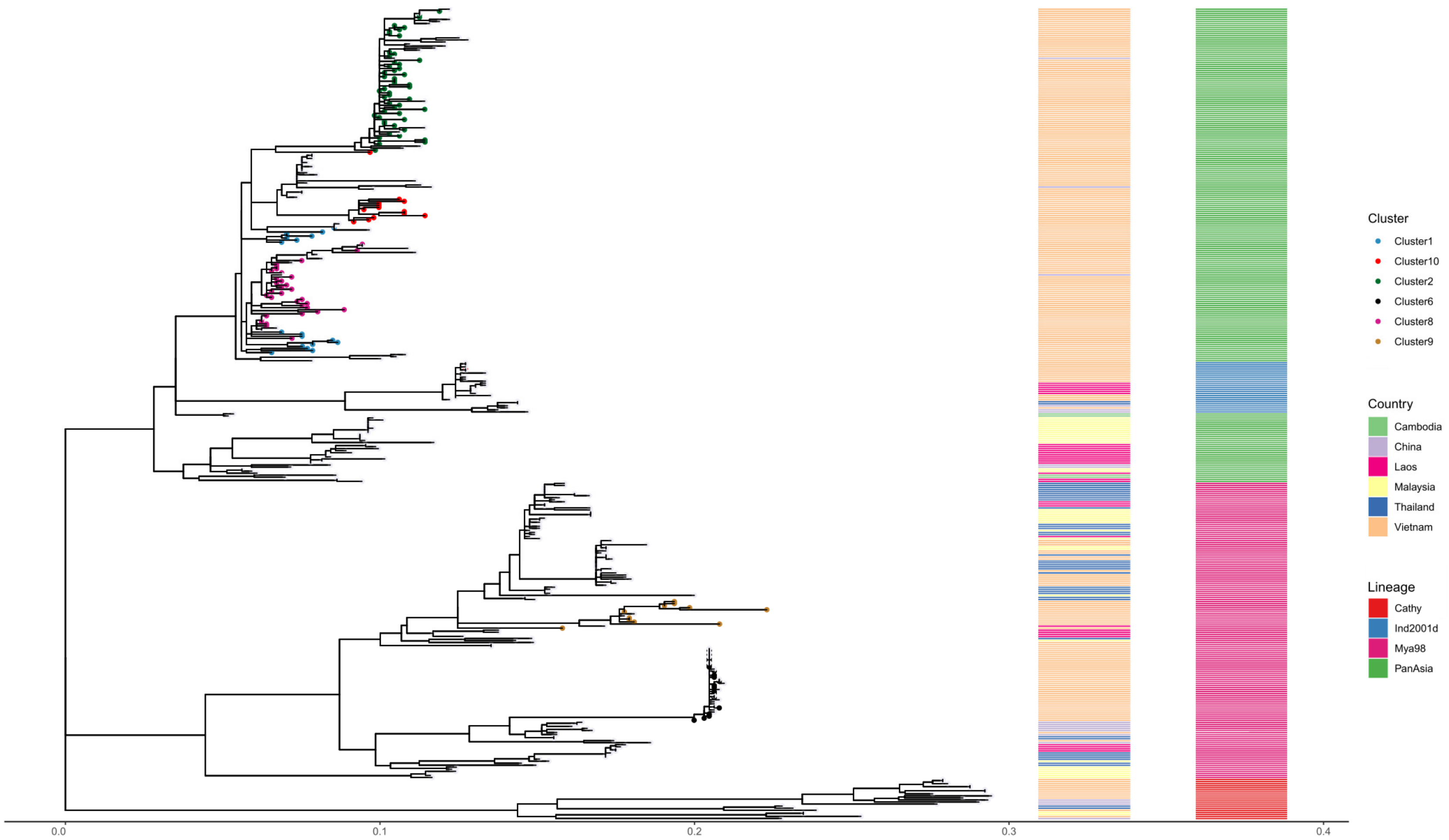

[Figure S1: Serotype O clusters shown in a maximum likelihood tree with other circulating FMD sequences obtained from South East Asian countries Cambodia, Laos, Malaysia, Thailand. Different node colors show the different clusters, only clusters with <10 sequences are labelled. Different bars show different lineages and the country where FMD sequence was isolated.]

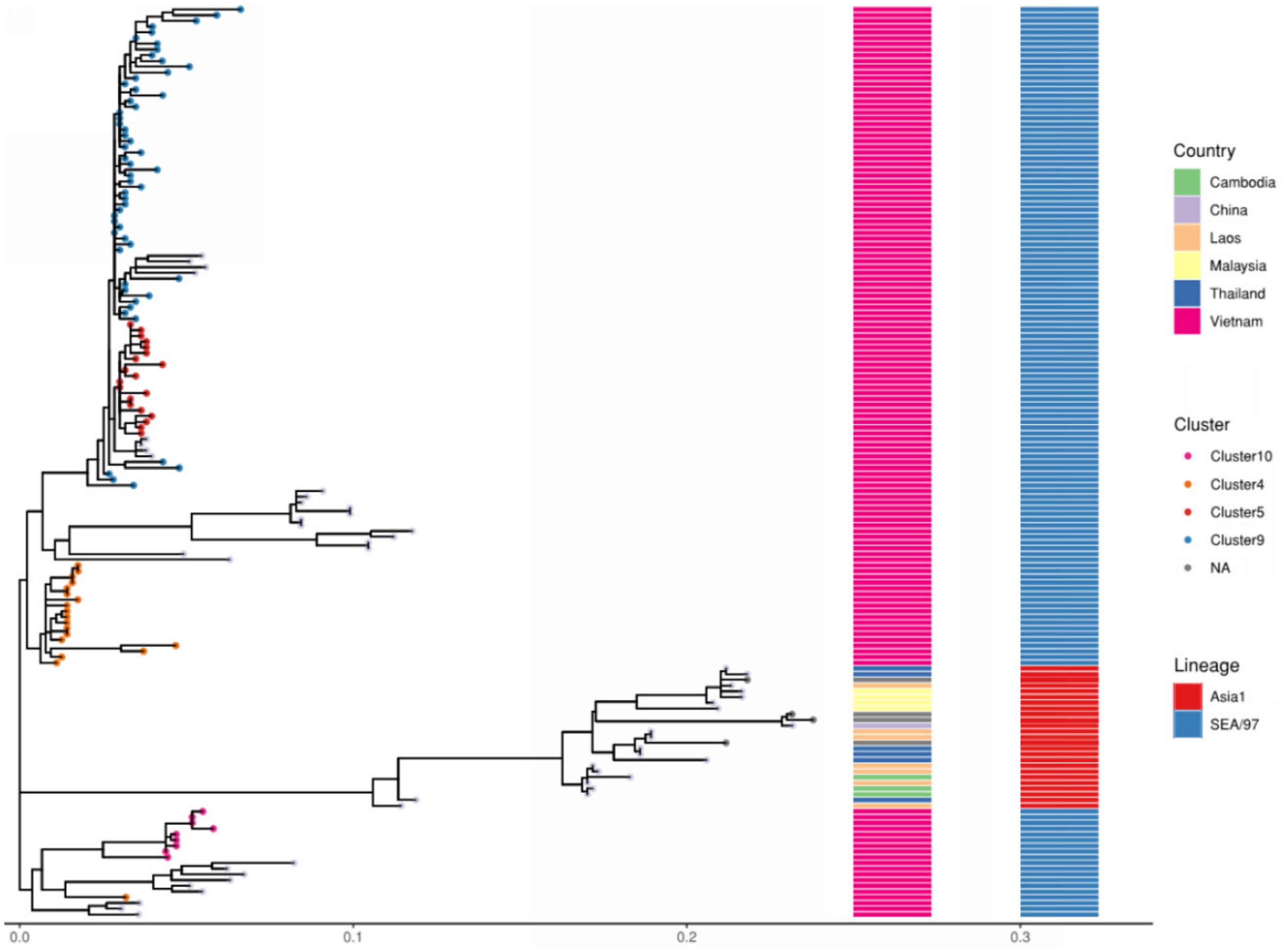

[Figure S2: Serotype A clusters shown in a maximum likelihood tree with other circulating FMD sequences obtained from South East Asian countries Cambodia, Laos, Malaysia, Thailand. Different node colors show the different clusters, only clusters with <10 sequences are labelled. Different bars show different lineages and the country where FMD sequence was isolated.]
